# Spatial Structure-Related Sensory Landmarks Recognition Based on Long Short-Term Memory Algorithm

**DOI:** 10.3390/mi12070781

**Published:** 2021-06-30

**Authors:** Yikang Wang, Jiangnan Zhang, Hairui Zhao, Mengjie Liu, Shiyi Chen, Jian Kuang, Xiaoji Niu

**Affiliations:** 1 School of Geodesy and Geomatics, Wuhan University, No. 129 Luoyu Road, Wuhan 430079, China; wangyikang@whu.edu.cn (Y.W.); jnzhang@whu.edu.cn (J.Z.); 2017301610177@whu.edu.cn (H.Z.); liumengjie@whu.edu.cn (M.L.); 2019302141282@whu.edu.cn (S.C.); 2 Department of Earth Science and Engineering, Imperial College London, London SW7 2BP, UK; 3 GNSS Research Centre, Wuhan University, No. 129 Luoyu Road, Wuhan 430079, China; kuang@whu.edu.cn

**Keywords:** machine learning, indoor localization, sensory landmark, Long Short-Term Memory (LSTM)

## Abstract

Indoor localization is the basis for most Location-Based Services (LBS), including consumptions, health care, public security, and augmented reality. Sensory landmarks related to the indoor spatial structures (such as escalators, stairs, and corners) do not rely on active signal transmitting devices and have fixed positions, which can be used as the absolute positioning information to improve the performance of indoor localization effectively without extra cost. Specific motion patterns are presented when users pass these architectural structures, which can be captured by mobile built-in sensors, including accelerometers, gyroscopes, and magnetometers, to achieve the recognition of structure-related sensory landmarks. Therefore, the recognition of these landmarks can draw on the mature methods of Human Activity Recognition (HAR) with improvements. To this end, we improved a Long Short-Term Memory (LSTM) neural network to recognize different kinds of spatial structure-related sensory landmarks. Labels of structural sensory landmarks were proposed, and data processing methods (including interpolation, filter, and window length) were used and compared to achieve the highest recognition accuracy of 99.6%.

## 1. Introduction

Indoor localization technology is the foundation of many Location-Based Services and has received increasing attention due to its wide range of applications [1,2]. Sensory landmarks in indoor environments can be leveraged to provide zero-cost absolute location information, hence improving indoor localization performance [3] and meeting the requirements for low-cost and high-accuracy positioning.

Sensory landmarks mainly have two roles in indoor localization: they provide absolute position calibration information and they assist in indoor mapping. Sensory landmarks are usually stable and have distinct characteristics in indoor environments. As a result, sensory landmarks can provide accurate absolute indoor positioning for users. Pedestrian Dead Reckoning (PDR) can only estimate the relative location and is prone to accumulated errors [4]. The absolute position provided by sensory landmarks can determine users’ current location and effectively correct this accumulated error [5], so as to form a low-cost, high-precision indoor positioning solution. Furthermore, indoor maps are essential components of indoor navigation and are usually required by indoor localization systems to display the user’s position. Indoor maps can be abstracted using a link-node model in which the pathways are the links and the intersections of the pathways are the nodes, such as corners, elevators, and stairs [6], i.e., structure-related sensory landmarks. Detecting pedestrians’ corresponding activities as they pass through the nodes can determine their location on the map. Based on the type of sensors used, sensory landmarks can be divided into active and passive sensory landmarks. Active sensory landmarks rely on signals sent by artificial active signal transmitters, such as (Global Navigation Satellite System) GNSS, Bluetooth, Wi-Fi, (Near-Field Communication) NFC, and Acoustic landmarks [7]. Passive sensory landmarks refer to the features existing in the indoor environment, such as landmarks related to the structure of the indoor space (such as escalators, stairs, and corners). Passive sensory landmarks can be determined using built-in mobile sensors, such as accelerometers [8], gyroscopes [9], and magnetometers [10]. Therefore, the passive sensory landmarks do not depend on the deployment of indoor environments and have the benefits of low cost and wide applicability. Sensory landmarks related to the spatial structures (such as escalators, stairs, and corners) are passive landmarks with the properties of stability, precision location, and ease of detection. 

The threshold method is a very typical method for identifying sensory landmarks by setting threshold values. For instance, using a threshold of angular velocity from the gyroscope to identify corner landmarks, and an acceleration threshold to detect stair landmarks [11]. There is a significant advantage of using a single sensor to recognize landmarks with obvious short-time motion patterns such as corners, but it is difficult for landmarks with continuous patterns such as escalators and stairs [3]. An alternative method is Human Activity Recognition (HAR), as the specific motion patterns are presented when users pass these architectural structures, which can be captured by built-in smartphone, including accelerometers, gyroscopes, and magnetometers, to achieve the recognition of structure-related sensory landmarks. Therefore, the recognition of the spatial structure-related sensory landmarks can be converted into HAR. 

The main works and contributions of this paper are summarized as follows:

1. We analyzed the sensory properties of the various spatial structure-related sensory landmarks. Based on this, (to meet the demand for low-cost and high-accuracy absolute indoor positioning), several sets of labels of sensory landmarks were designed, and the accuracy of recognition using each set of labels was tested separately. Based on the analysis and comparison, the optimum labels for each structure-related sensory landmark are proposed.
2. Based on the designed labels, we built an LSTM neural network to identify sensory landmarks and achieved 99.6% accuracy.
3. During the training phase, we explored the pre-processing parameters and hyperparameters that affect the final recognition accuracy and their implications.

The following sections of this paper are organized as follows: previous works are reviewed in Section 2; labels of structure-related sensory landmarks are proposed in Section 3; the experiments and results are presented in Section 4; and comparison and discussion with other works are introduced in Section 5. Finally, the conclusions are drawn in the Section 6. 

## 2. Related Works

One of the difficulties in the application of sensory landmarks in indoor positioning is landmark detection. Detection methods for active and passive sensory landmarks are quite diverse since the data used comes from different sensors and has different features.

The two main methods of recognizing active sensory landmarks are triangulation [12] and fingerprint matching [13]. Triangulation methods cannot achieve accurate positioning of sensory landmarks in complicated indoor environments due to multipath interference. The excessive amount of calculation of fingerprint matching and the high cost of placing essential signal emission sources makes it difficult to apply on a large scale [3]. In comparison, passive sensory landmarks neither rely on additional signal transmission devices nor interference by multipath, which is more valuable for adoption.

Previously, the detection of passive landmarks was mainly based on the data characteristics of a single sensor. For example, turning can be detected by a compass or gyroscope, and going up or down a set of stairs can be detected by an accelerometer or barometer [3]. However, when people pass by these landmarks, their motion changes will cause multiple built-in sensors to show distinctive features. The insufficient use of sensor data hindered the improvement of recognition accuracy. Previous multisensor fusion-based recognition methods have usually employed combined camera image recognition [14], resulting in excessive calculation and poor cost performance.

Since passive sensory landmarks are highly related to indoor spatial structures (such as escalators, stairs, elevators, revolving doors, etc.), people usually change their motion states when passing these landmarks, leading to features that can captured by built-in sensors such as IMUs. In terms of feature extraction, machine learning has been widely used to distinguish different data features due to its advantages of automation and accuracy.

Some previous studies applied machine learning, including traditional machine learning and deep learning methods, to sensory landmark recognition. A previous study [15] used the spatial pyramid kernel-based bag-of-words (SPK-BoW) histogram method to extract features and build image dictionaries, followed by combining the extreme learning machine (ELM) algorithm and sparse representation classifier (SRC) to train the neural network to achieve landmark recognition. However, it is difficult to practically apply because of the complexity and time-consuming of building image dictionaries and the high requirement for ambient light. The study by [16] used a deep learning approach to recognize sensory landmarks using data fragments of accelerometers, gyroscopes, magnetometers, and barometers, which achieved higher accuracy than traditional methods and machine learning methods. However, the temporal correlation between data fragments was not considered.

As the most popular method of processing data, the neural network has the features of large-scale parallel processing, distributed storage, elastic topology, and highly redundant nonlinear operations. Neural networks are trained with specific learning criteria to eventually obtain a suitable parametric model [17]. The use of neural networks can achieve a rational use of sensor data by forming a multisensor fusion recognition algorithm [18], to achieve better recognition results than SVM [19] and other methods. Long short-term memory (LSTM) is a temporal recurrent neural network architecture suitable for processing and predicting continuous time-series data [20]. Due to the strong temporal correlation of human activity states, the use of LSTM neural networks can efficiently identify the user’s motion states [4].

An important application of using machine learning to extract certain features of IMU data is HAR [7,21,22]. By acquiring and analyzing the accelerometer information of the mobile phone, different human activities can be determined according to the acceleration characteristics of each motion state. In addition, clustering [23], SVM [19,24], and deep learning [25] can also be used for HAR. The user’s activities can be efficiently identified using LSTM neural networks due to its specialty in time-series analysis [20], the strong temporal correlation of human activities [4] and the suitability of LSTM. The paper is precisely based the use of on LSTM-based HAR to identify and classify spatial structure-related sensory landmarks.

## 3. The Architecture of the System

**Figure 1:**
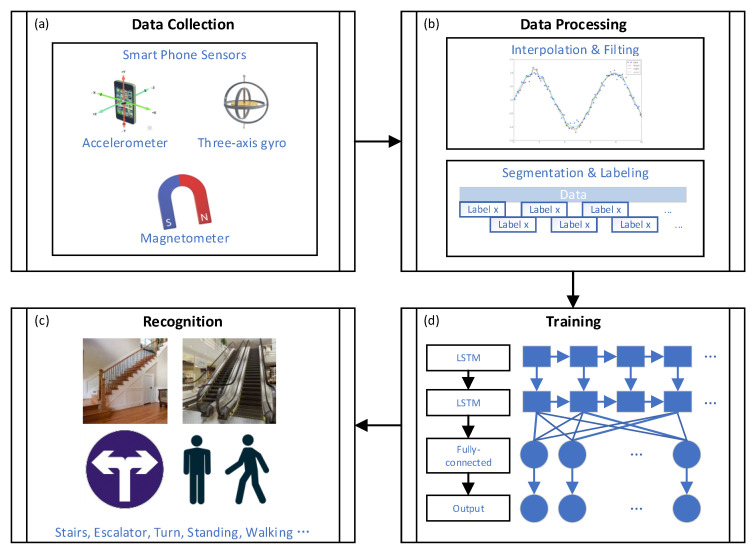
The schematic diagram of the system architecture (**a**) data collection (**b**) data processing (**c**) recognition and (**d**) training.

Figure 1 gives a schematic diagram of the system architecture for recognizing sensory landmarks, including (a) data collection, (b) data processing, (c) training, and (d) sensory landmark recognition. We use smartphones to collect data from the smartphone’s built-in sensors, including an accelerometer, a gyroscope, and a magnetometer. The data processing consists of two steps: aligning data frequencies of individual sensors by using interpolation and data filtering. Then, the timing-dependent data are sliced and labeled with the corresponding labels. We borrowed from HAR using a neural network containing a two-layer LSTM and a single fully connected layer for feature learning. Finally, the obtained model enables the recognition of spatial structure-related sensory landmarks such as stairs, escalators, and corners.

### 3.1. Label Design for Recognition of Sensory Landmarks

To meet the need for low-cost, high-precision indoor positioning, the sensory landmarks need to be stable on the time scale to avoid the high cost of updating landmark maps, be in small location areas with side length no longer than two meters to ensure precious positioning, and have unique sensory characteristics to enable high recognition accuracy.

However, HAR emphasizes states of human motion, such as walking, running, etc., and these motion states do not appear unique in the indoor environment. The accurate location of the user is not reflected by the motion state alone. Therefore, the labels used in HAR cannot simply be migrated to sensory landmark identification. A new set of labels needs to be designed so that the labeled sensory landmarks meet the needs of stability and location accuracy to provide the absolute location of landmarks. At the same time, it should have a high recognition accuracy rate, which is sufficient to support its follow-up use.

Besides, the sensors often used in HAR are those equipped in cell phones, including accelerometers, gyroscopes, barometers, and magnetometers, etc. Studies have shown that the more sensors combined for machine learning used in HAR, the better the recognition effect [16]. However, the latest cell phones, including the Huawei P series, are not always equipped with barometers, so they are not considered. Finally, the data from accelerometers, gyroscopes, and magnetometers are used in this paper.

The scenarios discussed in this paper include corners, escalators, and stairs. The starting point for designing the labels is the possible human motion states of the user in the scenario. In the above scenarios, the user may be walking or standing when not passing the sensory landmark. They will be identified as motion state labels for the states that do not pass by the sensory landmarks.

When we design the label corresponding to each landmark, we will mainly consider applying the definition to indoor localization and the accuracy of its recognition to the overall recognition. The impact of different label designs on recognition accuracy will be discussed in more detail in Section 4.

#### 3.1.1. Corners

In this paper, corners refer to areas where users take rapid turns, usually right-angle turns due to the distribution of indoor structures. The sensory landmark point is selected to appear at the location where the turn occurs. Corners are not designed to distinguish between left and the right turns since PDR can accurately determine the direction of rotation, so there is no need to increase the complexity of the neural network.

#### 3.1.2. Escalators

In the escalator scenario, the user’s movement is roughly divided into stages, including before entering the escalator, entering the escalator, on the escalator, leaving the escalator, and after leaving the escalator.

**Figure 2:**
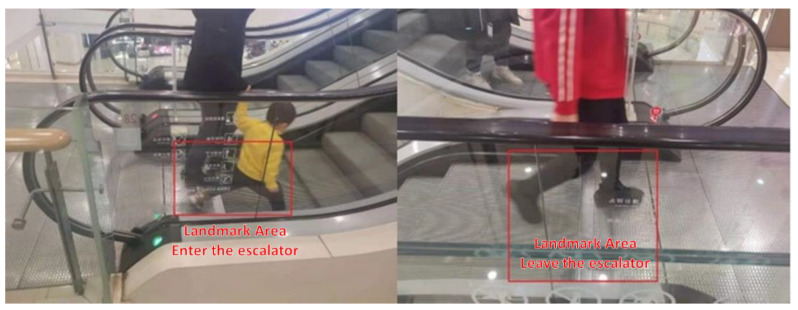
Diagram of the location of the entry and exit escalator landmarks.

The sensory landmark points that appear in the escalator scenario are the points of entry and exit from the escalator: specifically, the red rectangle position as marked in Figure 2, which shows the starting step of the escalator and the pedal of the escalator that the user steps on upon leaving the escalator.

The reason for not selecting the state as sensory landmarks are that the user’s position is relatively vague at that time, with a range of possible positions of one meter long and several meters wide, which is not suitable for accurate correction of the indoor position. The labels designed in the escalator scenario do not distinguish between up and down because the up and down process is more confusing, reducing the accuracy of identifying the corresponding label.

#### 3.1.3. Stairs

**Figure 3:**
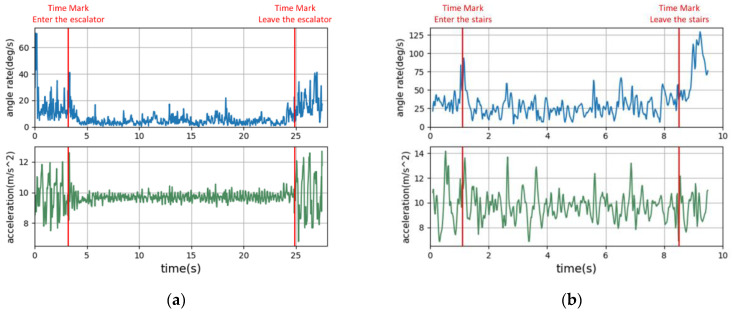
Data of accelerometer and gyroscope when the user passes: (**a**) the escalator; (**b**) the stairs.

Within the staircase, the user’s movement is approximately divided into before entering the staircase, up the staircase, on the staircase, and stepping off the staircase. The final choice is to take the whole section of the user’s movement between entering and leaving the staircase as the stairs label. It is different from the scheme adopted for escalators. The reasons for this are shown in Figure 3a,b, which are graphs of the variation of acceleration and gyroscope reading magnitude with the experiment time when passing the escalator and stairs in the experiment. In the figures, the first red line marks the moment when the user enters the escalator or stairs, and the second red line is the moment when the user leaves the escalator or stairs. As can be seen from the waveform, the states of the user entering, exiting, and staying on the escalator are well distinguished, while these states more confusing on stairs. This is because, in all three processes, the user’s performance is only one step up or down. At the same time, users can go up and down on the same flight of stairs, so it is not particularly meaningful to distinguish between up and down. However, distinguishing between up and down increases the complexity of the neural network. Additionally, there may be confusion between the upward and downward movements, which reduces the separate upward and downward processes’ recognition accuracy. Therefore, we do not distinguish between up and down in the staircase scenario.

However, to obtain landmarks with higher accuracy of location, we should process the location of entering the stairs and the location of leaving the stairs as sensory landmarks, and this can be achieved by subsequent processing to extract the first window of a section of a continuous stair state for entering the stairs, and the first window from the continuous stair state to another state for leaving the stairs as landmarks.

Finally, we designed the sensory landmark definition, as shown in Table 1.

### 3.2. Data Pre-Processing

#### 3.2.1. Interpolation and Filtering

Although usually gyroscopes, accelerometers, and magnetometers can acquire the same frequency data, the moments of collection are not all the same. Therefore, the acquired data need to be interpolated to have readings from all three sensors at the desired moment.

After interpolation, the sensor data are also filtered to exclude the presence of noise, such as high-frequency jitter in the data.

#### 3.2.2. Data Slicing and Labeling

**Figure 4:**
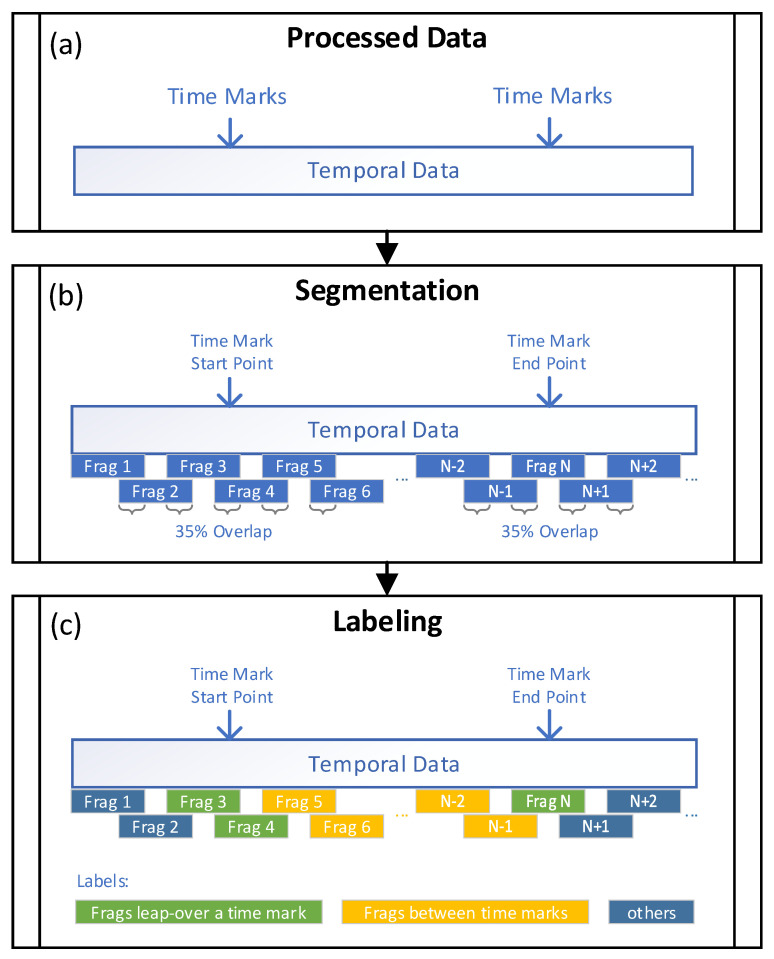
The diagram of segmentation and labeling. (**a**) Processed temporal data; (**b**) Data segmentation; (**c**) Data labeling.

The recognition of sensory landmarks cannot depend on a single data point. It is necessary to make judgments based on a segment of the user’s motion process. Slicing the entire user movement process is necessary to ensure that the neural network is given enough information at once without including excessive landmarks or user behaviors. In Figure 4, with the input of the processed data, the operations of slicing and labeling the data are shown.

We read in data from the accelerometer, gyroscope, and magnetometer in the phone for each segment of motion, using a sliding window for data slicing. Take the accelerometer data for example

(1) $$s_{x,i}^{f}=\left[f_{x,t}^{B},f_{x,t+1}^{B},\cdots ,f_{x,t+K-1}^{B}\right]\;s_{y,i}^{f}=\left[f_{y,t}^{B},f_{y,t+1}^{B},\cdots ,f_{y,t+K-1}^{B}\right]\;s_{z,i}^{f}=\left[f_{z,t}^{B},f_{z,t+1}^{B},\cdots ,f_{z,t+K-1}^{B}\right],$$

where $f^{B}$ is the acceleration in x, y and z axis under the body frame of the sensor, *t* is the start time of the time window *i*, and *K* is the length of the data sequence within a time window, determined by the time window length *T* and the sensor sampling frequency *v*:

(2) $$K=\frac{T}{\nu }$$

Similarly, for the gyroscope and the magnetometer, the data sequences $\left\{s_{i}^{gyro_{x}},s_{i}^{gyro_{y}},s_{i}^{gyro_{z}}\right\}$ and $\left\{s_{i}^{mag_{x}},s_{i}^{mag_{y}},s_{i}^{mag_{z}}\right\}$ can be obtained, respectively. For each independent motion process, the time length of each window sliding during the window sliding can be less than the time length of the window. This handles the fact that there is an overlap between the previous window and the next window, while there is no overlap between the corresponding independent motion processes. In this case, the amount of overlapping data between the two data windows before and after is expressed by the degree of overlap μ. The corresponding relationship between the overlap time length ΔT and the amount of overlapping sequence data ΔK is

(3) $$\Delta T=\mu T\;\Delta K=\frac{\Delta T}{\nu }$$

The specific labeling operation is according to the following rules. When the data fragment is located between the start and end time marks, the fragment is labeled as the intermediate process of the corresponding landmark (A3, A7). When the data fragment contains the time marks inside, it is labeled as the start and end moments of the sensory landmark (A1, A2, A4). When the data fragment lies outside the starting and ending time marks, it is marked as other control categories (A5, A6).

### 3.3. LSTM Network Architecture

After processing the data, it can be fed into the neural network. The neural network consists of a two-layer LSTM neural network and a one-layer fully connected layer. The first step of the neural network i the input vector of step $S_{i}$ is

(4) $$S_{i}={\left[s_{i}^{acce_{x}},s_{i}^{acce_{y}},s_{i}^{acce_{z}},s_{i}^{gyro_{x}},s_{i}^{gyro_{y}},s_{i}^{gyro_{z}},s_{i}^{mag_{x}},s_{i}^{mag_{y}},s_{i}^{mag_{z}}\right]}^{T}.$$

$S_{\;i}$ is a M×1 matrix of$\;M=N_{\;sensors}\times T/\;\Delta \;t$, where$\;N_{\;sensors}$ The input sensor data dimension is 9 in this study; *T* is the time window length, which is 2 s by default; and Δ t is the sampling interval, which is 0.02 s. The equation for forward propagation of the neural network through two LSTM layers and one fully connected layer can be simply expressed as

(5) $$a=f\left(W_{1}S_{i}+b_{1}\right),$$

where $W_{\;1}$ is a matrix of $\;N_{\;class}\times M$, and $N_{\;class}$ is the final number of classification labels, which is 7 in this study. a and$\;b_{\;1}$ are both$\;N_{\;class}\times 1$ matrices.

Similarly, the formula for backpropagation can be expressed as

(6) $${\hat{S}}_{i}=f^{\prime }\left(W_{2}a+b_{2}\right),$$

where $W_{\;2}$ is a matrix of $M\times N_{\;class}$ of the matrix, and$\;b_{\;2}$ is a$\;N_{\;class}\times 1$ matrix. In the training project, the neural network parameters are adjusted by backward pass so that the results are predicted with the highest accuracy, even though the error of the neural network$\;E_{\;i}$ is minimized.

(7) $$E_{i}=\overset{M}{\underset{j=1}{\sum }}{\left(S_{j}-{\hat{S}}_{j}\right)}^{2}$$

To avoid falling into local minima in training, we randomly initialized the neural network parameters several times, and the optimal accuracy is used as the result. After the training is completed, the neural network can identify the categories of sensory landmarks that users pass by. Algorithm 1 summarizes the method of recognizing sensory landmarks using LSTM neural networks.

**Algorithm 1:** LSTM Based Sensory Landmark Recognition.Input: labeled train data set $\left\{X^{Train},L^{Train}\right\}$ and unlabeled test data set $\left\{X^{Test}\right\}$ Output: predicted sensory landmark labels of the test data set $L^{Pre}$ 1 Collect data 2 Process data by interpolation, filtering, segmentation, and labeling 3 Initialize neural network parameters randomly 4 Train and compute the parameters 5 Use the trained neural network to predict the sensory landmark labels of the data set $L^{Pre}$

## 4. Experiments and Results

### 4.1. Experiment Description

A total of 13 different cell phones were used by 11 participants during data collection. Three or more locations in different indoor environments were selected for each scenario. During each collection, participants were required to keep their phones in hand and walk with them held to their chest level. They were also required to record information including the type of scenario, the type of landmark, and the moment of passing by the landmark. Cell phones were exchanged between participants after 20 sets of data were collected.

After the collection was completed, the acquired data were divided into a training set and a test set. To avoid overfitting, the test set contained all scenarios within it, while there was no overlap between the collected scenario locations and the training set. The final number of motion processes within the collected scenarios is shown in Table 2.

The collected data will be used in the method adopted in Section 3 where the parameters are shown in Table 3.

The processed data were fed into the LSTM neural network of the architecture, and the hyperparameters are shown in Table 4.

The total accuracy of the final LSTM model to identify the labels can achieve 99.6%, and the complete confusion matrix is shown in Table 5. The confusion between the individual labels is small, and a part of the state that originally went through the landmarks is identified as not going through the landmarks (i.e., corresponding to the A3, A5, A6 labels), which in practice is only a lost opportunity to accurately calibrate the location, rather than being identified as another landmark, in which the user position is updated to an incorrect location. This also has somewhat less impact on the accuracy of the final indoor positioning.

### 4.2. The Effect of Label Design on the Landmarks Recognition Accuracy

In this section, we will further discuss the overall accuracy of landmarks recognition under different label designs and analyze the causes.

#### 4.2.1. Escalator Scenario

**Figure 5:**
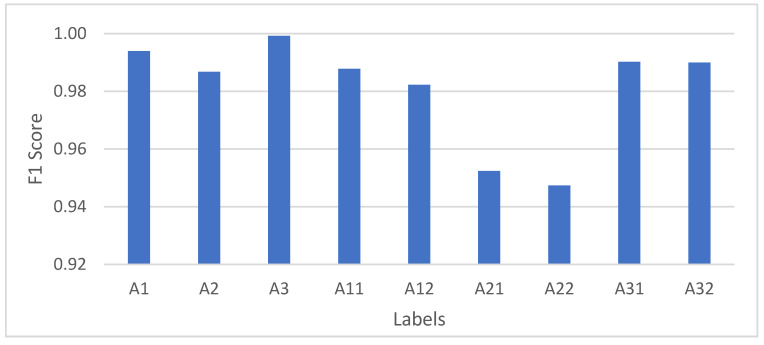
The F1 scores of the escalator landmarks before and after the splitting.

The focus of the escalator scenario is whether it is necessary to distinguish the ascending and descending escalator states. We use the original parameters based on the original experiment, and split the labels A1, A2, A3 in the original Table 4 into ascending and descending escalator to obtain A11, A12, A21, A22, A31, A32, where A11 represents the up row of the original label A1, A12 represents the down row of the original label A1, etc. for the rest. The hyperparameters were adjusted to obtain the highest accuracy, and the final confusion matrix is shown in Table 6. The comparison of the F1 scores of the escalator landmarks before and after the splitting is shown in Figure 5.

The confusion was created between the ascending and descending escalators, and it is very difficult to detect and correct through a short period of follow-up observation without the correction of the barometer. Therefore, it is not good practice to distinguish escalators ascending and descending.

#### 4.2.2. Staircase Scenario

**Figure 6:**
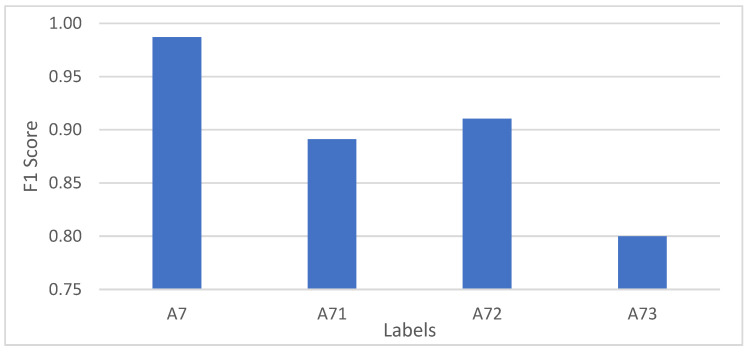
The F1 scores of the escalator landmarks before and after the splitting.

The focus of the staircase scenario is whether the user’s entry onto the staircase and their exit from the staircase need to be presented separately for identification. The A7 labels in the original Table 6 were split into ascending and descending escalators to obtain A71, A72, and A73, where A71 represents the user’s motion state between entering and leaving the stairs in the original A7 labeled stair landmarks, A72 represents entering the stairs, and A43 represents leaving the stairs. Based on the original experiments, the confusion matrix obtained by training using the original model parameters is shown in Table 7. The comparison of the F1 scores of the stair landmarks before and after the splitting is shown in Figure 6.

As can be seen, entering and leaving the stairs are not well distinguished from the actions of users when on the stairs. As the starting point and focus of a section of action, the wrong identification of entering and leaving the escalator label has a very bad effect, so it is not desirable to identify entering and leaving the stairs separately.

### 4.3. The Parameters that Affect the Final Recognition Accuracy

In this section, we will discuss the effect of some parameters on the total accuracy. Table 8 shows the effect of data preprocessing parameters on the total accuracy. The effect of model parameters on the total accuracy is given in Table 9.

## 5. Discussion

Given that the focus of the paper is on the recognition of sensory landmarks using LSTM methods, we focus our comparison on the accuracy of the different methods used to recognize sensory landmarks.

The research presented in [1] focuses on the refinement of the CNN network to finally arrive at a maximum accuracy, and also replicate the methods of DT, LDA, KNN, and SVM to finally arrive at their related accuracy of recognizing sensory landmarks.

**Figure 7:**
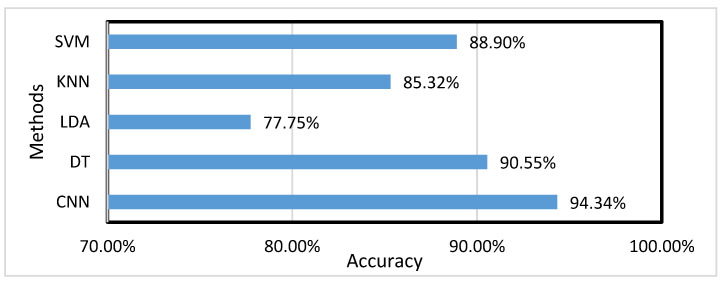
Accuracy of different methods for identifying sensory landmarks.

Since the types of identified sensory landmarks mentioned in [16] are roughly similar to our identified sensory landmarks, we can compare them by their total accuracies. Figure 7 shows the comparison of our LSTM with CNN and other traditional machine learning algorithms. In the table, we can clearly see that the neural network outperforms traditional machine learning, while the LSTM neural network outperforms the CNN network in terms of recognition accuracy.

**Figure 8:**
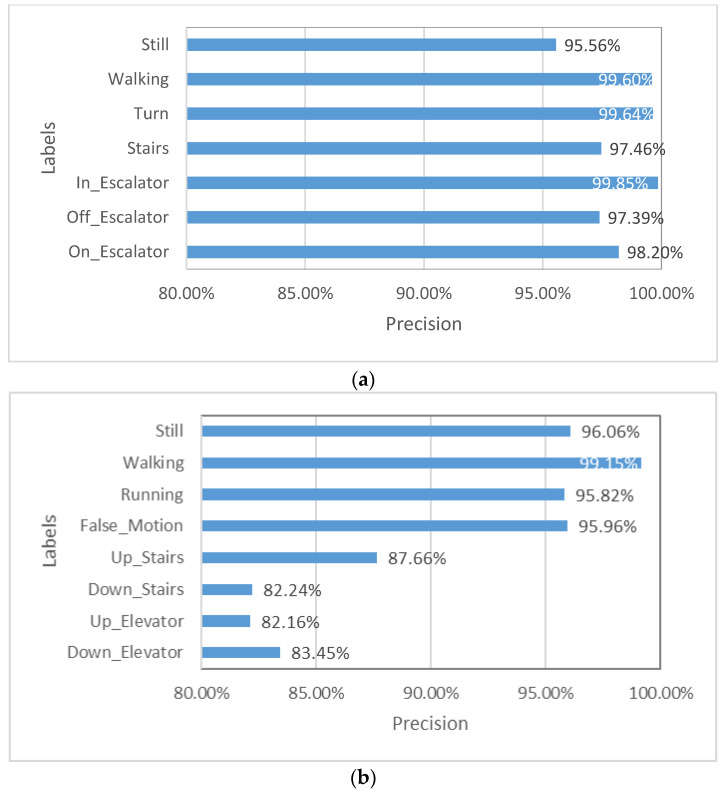
Accuracy of each label in **(a)** LSTM; **(b)** CNN.

Figure 8 shows the accuracy of each label using CNN [1] and LSTM. Although the labels are not exactly the same, the forms of the data are close, being data fragments with labels, and the numbers of labels are close, so it can reflect the models’ ability to recognize landmarks. CNN and LSTM perform similarly on the nonlandmark labels of “still” and “walking”, while LSTM performs better on most landmark labels. Therefore, LSTM is more capable of recognizing landmarks than CNN.

This paper presents an algorithm for the recognition of signal landmarks that achieves a high level of accuracy. This method can be applied in real-time locating systems (RTLS) by providing absolute position information for PDR. The indoor maps are not necessary, but the location of sensory landmarks should be known previously—whether by advanced manual tagging or crowdsourced collection. The performance required for this method is very low and suitable for smartphones since the period of computing equals the length of a time window (1—overlap), which is 3 s ×(1−25%)=2.25 s.

We have only collected data on phones held at chest level so far. As the application of the proposed methods relies on PDR, and the basic assumption of PDR is that the phone is oriented parallel to the direction of pedestrian walking. Additionally, our method requires reading data from multiple sensors on the phone, and most smartphones, for privacy reasons, only allow the app to read sensor data when it is running in the foreground and with the screen on. Therefore, holding the phone at chest level should be the basic situation of this method. By introducing a coordinate transformation, the improved PDR can also be used when the phone orientation is not parallel to the pedestrian walking. In this situation, our method can keep working with the same coordinate transformation. For more complex situations such as swinging arms, recognition can be achieved by collecting data and retraining the neural network.

Although this method has a high accuracy of 99.6%, it still suffers from a low probability of misclassification, and reducing the impact of false recognition in pedestrian indoor localization applications will be the focus of our future work. Based on the fact that two neighboring landmarks are usually located far apart in real environments, we plan to introduce a continuous judgement to find and correct miscalculations that conflict with the previous and subsequent results. Alternatively, faulty recognition results can also be identified by combining the PDR predicted user location and the sensory landmarks database.

## 6. Conclusions

Based on the data from built-in smartphone, including an accelerometer, a gyroscope, and a magnetometer, the recognition of structure-related sensory landmarks, such as stairs, escalators, and corners, is achieved by a double-layer LSTM neural network. Through exploring reasonable label designs and processing sensory data by interpolation, filtering, and segmentation, the accuracy of recognition achieved is as high as 99.6%, which is better than many previous methods. The accurate recognition of sensory landmarks can provide location correction information for PDR without active signal transmitting devices, establishing a strong basis of low-cost and high-precision indoor localization.

## Figures and Tables

**Table 1:** Table of labels.

No.	Name of Labels	Explanation
A1	ON_ESCALATOR	The landmark where the user enters the escalator, before the user is in the walking state, afterward the in_escalator state
A2	OFF_ESCALATOR	The landmark where the user leaves the escalator, before the user is in_escalator state, afterward in the walking state
A3	IN_ESCALATOR	The user is in an upward or downward movement on the escalator
A4	TURN	Turning landmarks during the user’s travel, before and after the walking state
A5	WALKING	The user’s walking forward state
A6	STILL	The user’s stationary state
A7	STAIRS	User status on stairs up/down

**Table 2:** Amount of data collected.

Scenarios	Training Set	Test Set
escalator	946	217
stairs	833	76
corners	3108	480
still	594	113

**Table 3:** Data pre-processing parameters.

Parameters	Parameter Description	Parameter Values
T	Length of a time window	3 s
Interpolation method	Interpolation method and order	Lagrange 4th order interpolation
Filtering method	The method used for filtering (frequency)	Low-pass filtering 0–3 hz
Μ	The overlap of time windows	25%
*ν*	The sampling frequency of sensors	50 hz

**Table 4:** Selected hyperparameters.

Parameters	Parameters Description	Parameter Values
M	Dimensions of input data	900
batch_size	Number of samples selected for one training session	1500
$N_{sensors}$	Number of dimensions of the sensor data used	9
$N_{class}$	Number of labels to be identified	7
$N_{hidden}$	Hidden layer num of features	40

**Table 5:** The confusion matrix.

	Prediction	A1	A2	A3	A4	A5	A6	A7
Truth								
**A1**		164	0	0	1	1	0	0
**A2**		0	149	0	0	3	0	0
**A3**		0	1	1364	0	0	0	0
**A4**		0	0	0	279	0	0	0
**A5**		0	0	0	0	493	0	1
**A6**		0	0	1	0	0	43	0
**A7**		0	0	0	0	2	0	115

**Table 6:** Confusion matrix for escalator differentiation between ascending and descending escalators.

	Prediction	A11	A21	A31	A4	A5	A6	A7	A12	A22	A32
Truth											
**A11**		81	0	0	0	0	0	0	0	0	0
**A21**		0	70	0	0	1	0	1	0	4	0
**A31**		0	0	661	0	0	0	0	0	0	7
**A4**		0	0	0	278	0	0	0	1	0	0
**A5**		0	0	0	0	493	0	1	0	0	0
**A6**		0	0	0	0	0	43	0	0	0	1
**A7**		0	0	0	0	2	0	115	0	0	0
**A12**		2	0	0	0	0	0	0	83	0	0
**A22**		0	1	1	0	2	0	0	0	72	0
**A32**		0	0	5	0	0	1	0	0	0	691

**Table 7:** Confusion matrix for escalator differentiation between ascending and descending escalators.

	Prediction	A1	A2	A3	A4	A5	A6	A71	A72	A73
Truth										
**A1**		223	0	3	0	9	0	1	1	1
**A2**		0	237	5	1	3	1	2	4	0
**A3**		4	3	2499	1	0	0	0	0	0
**A4**		0	1	1	675	5	0	0	1	1
**A5**		1	1	0	0	848	0	3	1	0
**A6**		0	0	1	0	0	63	0	0	0
**A71**		1	0	0	0	10	1	221	0	7
**A72**		0	1	0	4	0	0	8	122	0
**A73**		0	0	0	3	9	1	21	4	94

**Table 8:** The effect of data pre-processing parameters on overall accuracy.

Parameters	Parameter Effect on the Overall Accuracy
T	When the window length is below 2 s, the accuracy of identifying stairs and escalators drops significantly, by more than 40%. This indicates that when the window length is insufficient, a window does not contain enough information to recognize the user’s movement on an escalator. When exceeding 2 s, the recognition accuracy increases with increasing window length.
Interpolation method	The impact is not significant. The impact on the total accuracy is no more than 1%.
Filtering method	Using low-pass filtering for accelerometers can result in a significant improvement in accuracy, by about 9 percent. Using low-pass filtering for gyroscopes, on the other hand, can lead to a decrease in recognition accuracy.
μ	The window overlap has little effect on the total accuracy. The total accuracy impact is around 2%.

**Table 9:** The effects of hyperparameters on overall accuracy.

Parameters	Description of Parameters
$N_{hidden}$	When it increases, the total accuracy of the model also increases. When the value is from 12 to 36, the improvement is more significant, and when it is greater than 36, the improvement effect slows down.
Neural Network structure	Adding the fully connected layer after the current neural network structure has no significant effect on the overall accuracy. The overall effect does not exceed 1.
